# Supplementary material for: Construction of Two Independent RAB Family-Based Scoring Systems Based on Machine Learning Algorithms and Definition of RAB13 as a Novel Therapeutic Target for Hepatocellular Carcinoma
Source: Int J Mol Sci. 2023 Feb 22;24(5):4335. doi: 10.3390/ijms24054335 (PMC10001462; doi:10.3390/ijms24054335)
Supplement: Supplementary file 1 [file ijms-24-04335-s001.zip › Supplementary_Material.pdf]

Supplementary Material

1 Supplementary Figures

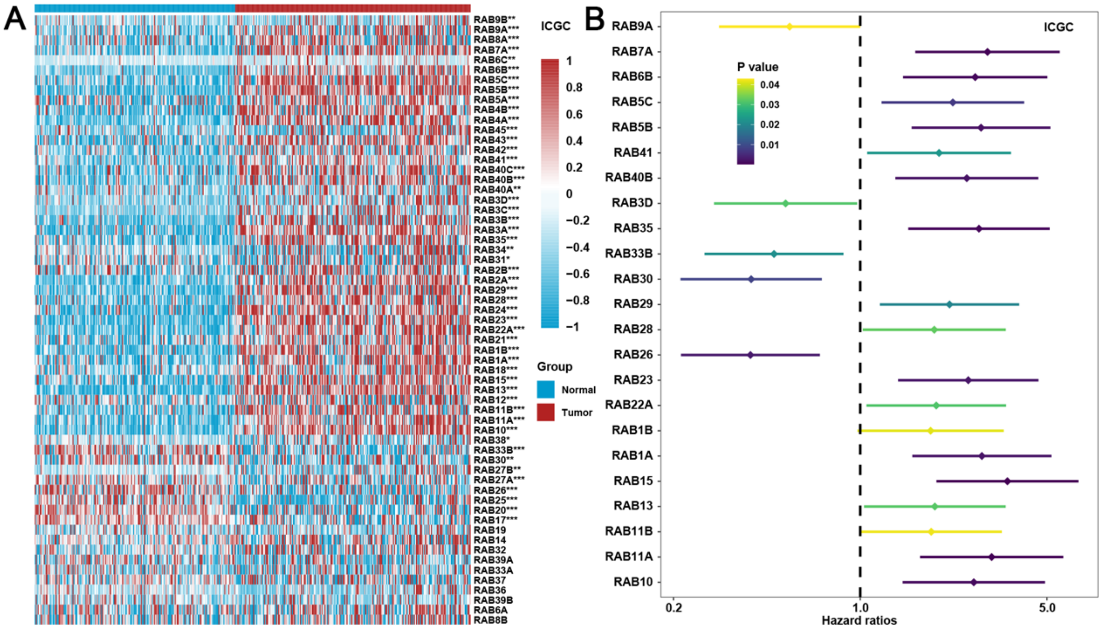

Figure S1. (A) Heatmap of RAB family genes in HCC and normal liver (Normal) tissues from the ICGC cohort. (B) Survival analyses for RAB family members of the ICGC cohort.

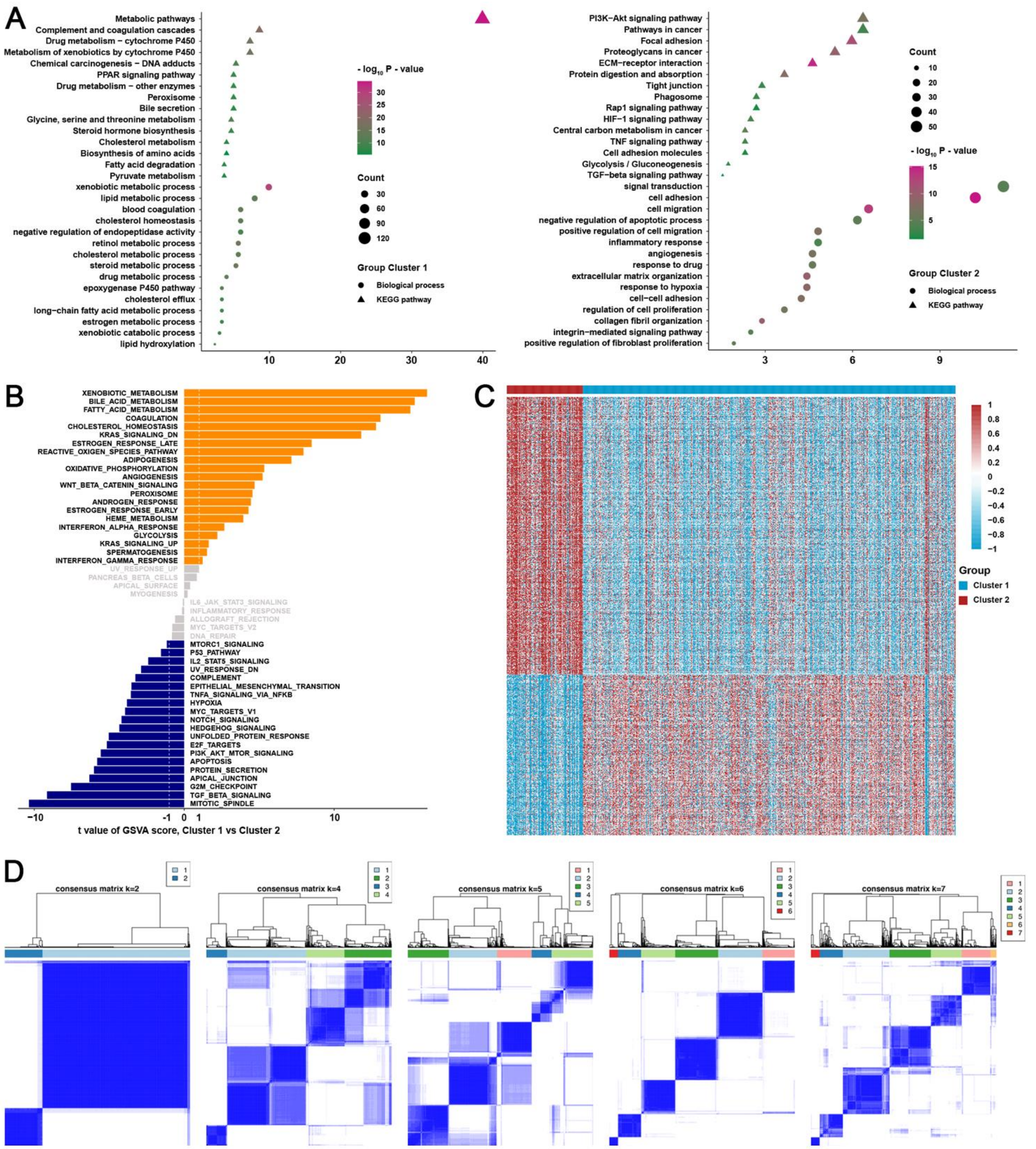

Figure S2. (A) KEGG signaling pathway enrichment analysis for two RAB clusters in the pooled HCC cohort. (B) GSVA annotations for two RAB clusters in the pooled HCC cohort. (C) Heatmap exhibiting the differentially expressed genes in the two RAB clusters. (D) Consensus matrices of the pooled HCC cohort for k = 2, 4, 5, 6 and 7.

2 Supplementary Tables

2.1 Table S1: RAS oncogene GTPases (RAB) family from HGNC database.

2.2 Table S2: The AUC values of RAB family members in TCGA-LIHC and ICGC-LIHC datasets.

2.3 Table S3: The RAB-associated gene signatures contains 830 genes.

2.4 Table S4: Coefficients of the genes involved in RAB risk score.

2.5 Table S5：Sequences of siRNAs in this study.

**Primer names**	**Sequences**
siRAB13-1 sense	5’-CGACAAUUCUGGUUACAAAGG-3’
siRAB13-1 anti-sense	5’-UUUGUAACCAGAAUUGUCGUG-3’
siRAB13-2 sense	5’-GAGCGGUUCAAGACAAUAACU-3’
siRAB13-2 anti-sense	5’- UUAUUGUCUUGAACCGCUCUU-3’

2.6 Table S6: The qRT-PCR primers used in this study.

Primer names	Species	Sequences
β-actin forward	Human	5’-GAAGATCAAGATCATTGCTCC-3’
β-actin reverse	Human	5’-TACTCCTGCTTGCTGATCCA-3’
RAB13 forward	Human	5’-TTGCAGAGGACAACTTCAACAA-3’
RAB13 reverse	Human	5’-CTATATCCACAGTGCGGATCTTG-3’

2.7 Table S7: Primary antibodies used in this study.

Antigens	Manufacturer	Catalog Number	Application
RAB13	ABclonal	A10571	1:800 for WB

PI3K	Abcam	Ab191606	1:800 for WB
p-PI3K	Abcam	Ab182651	1:500 for WB
AKT	Abcam	Ab179463	1:800 for WB
p-AKT	Abcam	Ab192623	1:500 for WB
IRF4	Proteintech	66451-1-Ig	1:800 for WB
N-cadherin	HUABIO	ET1607-37	1:1000 for WB
E-cadherin	HUABIO	ET1607-75	1:1000 for WB
Vimentin	Proteintech	10366-1-AP	1:1000 for WB
Slug	Proteintech	12129-1-AP	1:1000 for WB
JAK2	HUABIO	ET1607-35	1:800 for WB
p-JAK2	HUABIO	ET1607-34	1:500 for WB
STAT3	HUABIO	ET1607-38	1:800 for WB
p-STAT3	HUABIO	ET1607-39	1:500 for WB
CDK1	Proteintech	19532-1-AP	1:800 for WB
CDK4	Proteintech	11026-1-AP	1:800 for WB
GAPDH	Proteintech	60004-1-Ig	1:2500 for WB

WB, western blotting.

2.8 Table S8: The gene sets used for estimating the enrichment score in pathway and biological process activity.
